# Slowing Replication in Preparation for Reduction

**DOI:** 10.1371/journal.pgen.1002715

**Published:** 2012-05-17

**Authors:** Katherine S. Lawrence, JoAnne Engebrecht

**Affiliations:** Department of Molecular and Cellular Biology, University of California Davis, Davis, California, United States of America; The University of North Carolina at Chapel Hill, United States of America

Meiosis reduces the ploidy of the genome to generate haploid gametes for sexual reproduction. As gametes are portals for the generational transfer of genetic material, it is imperative that the genome is copied accurately and that chromosomes segregate equally into each haploid gamete. Proper chromosome segregation requires the formation of specialized chromosome axes to create and maintain an environment competent for double-strand break (DSB) formation and homologous recombination. Although the fundamental copying mechanism appears to be identical in mitosis and meiosis, the S phase that precedes meiosis (meiS) is at least twice as long as mitotic S phase (mitS) [1]–[4]. The underlying basis for an extended S phase prior to meiosis has, until now, been mysterious. While it is postulated that meiS length contributes to the dramatic chromosome reorganization that occurs during meiotic prophase (Figure 1), there is conflicting data concerning the interdependencies of meiS, chromosome morphogenesis, and DSB formation [5]–[9]. In this issue of *PLoS Genetics*, Blitzblau et al. [10] use innovative genome-wide approaches in yeast to elucidate mechanisms underlying meiS length and provide insight into the relationship between DNA replication and meiotic prophase events.

**Figure 1:**
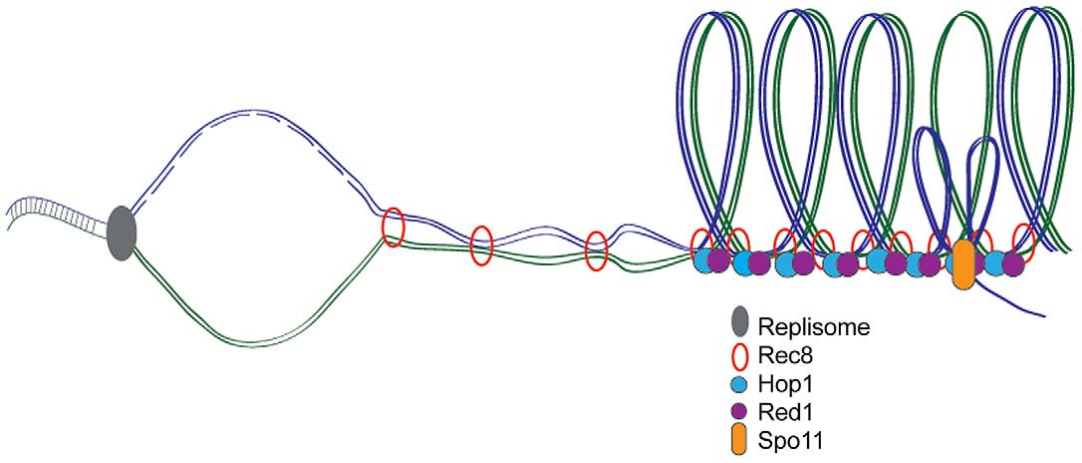
Meiotic DNA replication, chromosome axes, and DSB formation. During meiosis, DNA is replicated and meiosis-specific cohesin Rec8 holds sister chromatids together while axis proteins Red1 and Hop1 associate to form the loop axis structure. Endonuclease Spo11 creates DSBs required for homologous recombination and crossover formation at the axis where single-stranded DNA is exposed to facilitate homology search.

## Delayed Origin Firing Slows Meiotic S Phase

To compare mitS and meiS, Blitzblau et al. performed genome-wide chromatin immunoprecipitation (ChIP) to identify Mcm replicative helicase binding sites in conjunction with microarray analysis to monitor actively replicating origins. Importantly, synchrony was achieved by taking advantage of an ATP analog–sensitive mutant of Ime2, a kinase essential for DNA replication and meiotic entry, allowing for conditional inactivation. The authors found that Mcm bound to a significant fraction of the same origins in both mitosis and meiosis; the few that differed were located near cycle-specific, actively transcribed genes, consistent with studies suggesting competition between replication and transcription machinery [11]. Thus, the small differences in Mcm occupancy are unlikely to account for the timing differences between mitS and meiS. In contrast, while origins fired in the same relative order, as reported previously for a single chromosome [12], replication initiation at a significant fraction of origins was delayed in meiS compared to mitS.

Blitzblau et al. identified early meiS replication sites by analyzing replication in the presence of the ribonucleotide reductase inhibitor hydroxyurea (HU), which depletes nucleotide pools and prevents late origins from firing [13]. While all early replication meiS sites were shared in mitS, only 38% of early mitS sites initiated replication in meiS, consistent with the delay in origin firing and extended meiS length.

In the course of these experiments, the authors found that meiS is more sensitive to HU and has more robust checkpoint signaling than mitS. To explore whether this is a consequence of reduced nucleotide pools, Blitzblau et al. treated meiotic cells with HU in the absence of ribonucleotide reductase inhibitor SML1 [14]. The number of early firing origins increased, but not to the level utilized in mitotic cells, suggesting nucleotide depletion contributes to delayed origin firing and meiS timing.

## MeiS, Chromosomal Axis Formation, and DSB Competency Can Be Uncoupled

It has been proposed that meiS is slowed to facilitate the elaboration of meiosis-specific chromosomal structures and DSB formation (Figure 1) [5]. Blitzblau et al. probed the relationship between axis and DSB formation and meiS timing by examining early origin firing in the presence of HU in axis mutants (*rec8Δ*) or in cells that do not induce DSBs (*spo11Δ*). No significant differences between the replication profiles were observed, suggesting that loading of meiosis-specific proteins and break formation do not regulate meiotic replication timing.

To test whether DNA synthesis, in turn, affected axis and DSB formation, the authors examined the association of meiosis-specific axis components (Red1 and Hop1) following replication arrest by HU, depletion of Mcm loading factor Cdc6, or in the absence of B-type cyclins. In all situations, axis proteins were loaded onto chromosomes, suggesting replication is not an absolute prerequisite for axis formation. Blitzblau et al. also found that the *cdc6* mutant was competent for DSB formation, indicating that breaks can occur on unreplicated chromosomes. This is in contrast to a previous study that found that delaying replication delayed break formation [8]. The discrepancy is most likely due to shared regulatory components between DNA replication and DSB formation that are disrupted in *cdc6* mutants.

## Slow S, Replication Fidelity, and Metazoans

The current study provides strong evidence that in *S. cerevisiae* there is reduced replication capacity during meiS, at least in part due to limiting nucleotide pools. This manifests as delayed firing of a significant fraction of origins and extended S phase; a similar pattern of origin firing has been observed in *S. pombe* [2]. In both of these organisms, meiosis is initiated by starvation conditions, which presumably alters the activity of cell cycle components as well as decreases nucleotide pools. The applicability of this finding to metazoans, where meiS is also extended [1], [3] is unknown as there is no evidence that germ cells in multicellular organisms experience limiting nutrients. The authors suggest that extended S phase increases replication fidelity; however, in metazoans, germ cells can undergo multiple rounds of DNA replication prior to meiotic entry; these replications must also occur accurately. While no direct comparison of error rate between mitS and meiS has been performed, DNA polymerase mutants that have an inherently lower misincorporation frequency also have reduced processivity [15], suggesting that slowed replication could increase fidelity. However, this would manifest in reduced fork rates, something not directly addressed in this study. Perhaps the enhanced checkpoint signaling observed in meiS reflects more robust surveillance mechanisms that promote fidelity irrespective of nucleotide pools. Future work examining nucleotide pools, replication fidelity, and checkpoints may shed light on the significance of extended meiotic S phase in both single-celled and multicellular organisms.

While the authors conclusively demonstrate that chromosomal axis and DSB formation can occur in the absence of DNA replication and do not directly impinge on replication timing, these processes are nonetheless linked and occur successively in wild-type cells (Figure 1). Thus there is still much to be learned about how DNA replication is modified in meiosis to ensure the transfer of genetic material from one generation to the next.
